# Continuous Glucose Monitoring-Derived Metrics and Capillary Vessel Density in Subjects with Type 1 Diabetes without Diabetic Retinopathy

**DOI:** 10.1155/2023/9516059

**Published:** 2023-04-15

**Authors:** Antonio Cutruzzolà, Adriano Carnevali, Valentina Gatti, Giovanni Latella, Luca Lamonica, Flavia Oliverio, Massimo Borelli, Martina Parise, Sergio Di Molfetta, Vincenzo Scorcia, Concetta Irace, Agostino Gnasso

**Affiliations:** ^1^Department of Clinical and Experimental Medicine, University Magna Graecia, Catanzaro, Italy; ^2^Department of Medical and Surgical Sciences, University Magna Graecia, Catanzaro, Italy; ^3^UMG School of Ph.D. Programmes Life Sciences and Technologies, University “Magna Græcia”, Catanzaro, Italy; ^4^Department of Health Science, University Magna Graecia, Catanzaro, Italy; ^5^Department of Precision and Regenerative Medicine and Ionian Area, Section of Internal Medicine, Endocrinology, Andrology, and Metabolic Diseases, University of Bari Aldo Moro, Bari, Italy

## Abstract

Optical coherence tomography angiography (OCTA) is an innovative and reliable technique detecting the early preclinical retinal vascular change in patients with diabetes. We have designed our study to evaluate whether an independent relationship exists between continuous glucose monitoring (CGM)-derived glucose metrics and OCTA parameters in young adult patients with type 1 diabetes without diabetic retinopathy (DR). Inclusion criteria were age ≥ 18 years, diagnosis of type 1 diabetes from ≥ 1 year, stable insulin treatment in the last three months, use of real-time CGM, and CGM wear time ≥ 70%. Each patient underwent dilated slit lamp fundus biomicroscopy to exclude the presence of DR. A skilled operator performed OCTA scans in the morning to avoid possible diurnal variation. CGM-derived glucose metrics from the last 2 weeks were collected through the dedicated software during OCTA. Forty-nine patients with type 1 diabetes (age 29 [18; 39] years, HbA1c 7.7 ± 1.0%) and 34 control subjects participated in the study. Vessel density (VD) of the whole image and parafoveal retina in the superficial (SCP) and deep capillary plexus (DCP) was significantly lower in patients with type 1 diabetes compared to controls. The coefficient of variation of average daily glucose, evaluated by CGM, significantly correlated with foveal and parafoveal VD in SCP and with foveal VD in DCP. High glucose variability might be responsible for the early increase of VD in these areas. Prospective studies may help understand if this pattern precedes DR. The difference we detected between patients with and without diabetes confirms that OCTA is a reliable tool for detecting early retinal abnormalities.

## 1. Introduction

Optical coherence tomography angiography (OCTA) is an innovative and reliable technique providing rapid and noninvasive images of the retinal and choroidal vasculature and structure. Specifically, OCTA allows the evaluation of the superficial capillary plexus (SCP) slab, which is the capillary network embedded in the ganglion cell layer and/or the nerve fiber layer; the deep capillary plexus (DCP) slab that consists of the capillary network in the inner nuclear layer; and the choriocapillaris slab. The OCTA has recently been introduced as an additional modality detecting the early preclinical retinal vascular change in patients with diabetes. This innovative technique details vascular morphology, such as branching, tortuosity, and vascular density [1–3]. The OCTA features are closely consistent with histology and correlate with other in vivo imaging [4–6]. Vascular abnormalities detected by OCTA have been associated with the early treatment diabetic retinopathy study (ETDRS) severity scale, both in proliferative and nonproliferative diabetic retinopathy (DR). For example, patients with mild nonproliferative DR have mild loss of vessel density (VD).

In contrast, those with moderate and severe nonproliferative DR have a more marked loss of VD, abnormal vessel caliber, and areas of capillary nonperfusion [7, 8]. Of note, OCTA abnormalities, including enlargement of the foveal avascular zone (FAZ), loss of VD, and impairment of the choriocapillaris flow, may occur even in the absence of the typical signs of DR [3, 8–10]. The relationship between indices of glycemic control, such as fasting blood glucose, glycated hemoglobin (HbA1c), and VD, is controversial. Indeed, some studies found a significant inverse correlation, and others did not find any association [10–14]. Conversely, hyperlipidemia, smoking, renal impairment, and systolic blood pressure are predictors of VD loss [10, 15].

Following the advent of continuous glucose monitoring (CGM), new indices of glucose control and variability (GV) have entered the clinical practice, including time spent in the target range (TIR), time spent below the target range (TBR), time spent above the target range (TAR), 24-hour average glucose, glucose management indicator (GMI), coefficient of variation (CV), and standard deviation (SD) of average glucose. The suggested target glucose range is 70-180 mg/dL, and the optimal percentage of time to spend in the target range is ≥70%. GV refers to any fluctuation in blood glucose levels; the preferred metric to assess GV is the CV, which should not exceed 36% [16]. The interest in CGM as a possible standard tool to evaluate glycemic control in clinical research is growing. Indeed, the new indices, unlike HbA1c, give information on the burden of hyperglycemia, hypoglycemia, and GV, which can be considered possible markers and predictors of diabetes complications.

To our knowledge, only one paper by Pilotto et al. evaluated the association between retinal changes detected by OCTA and CGM-derived glucose metrics. In a cohort of adolescents with type 1 diabetes, with or without DR, the authors found that vascular density in the intermediate capillary plexus is inversely related to average glucose and SD and directly related to TIR [17].

Given the ability of OCTA to find early retinal abnormalities in subjects with type 1 diabetes, we have designed our study to evaluate if an independent relationship between CGM-derived glucose metrics and OCTA parameters exists in young adult patients with type 1 diabetes without DR.

## 2. Methods

### 2.1. Study Design and Patients

The current research is a cross-sectional study enrolling consecutive patients with type 1 diabetes, regularly attending the diabetes care center at the Magna Graecia University teaching hospital, Catanzaro. The local ethical committee was informed about the aim of the research, which was conducted according to the principles of good clinical practice. Patients who met the inclusion and exclusion criteria and gave informed consent were enrolled in the study. Inclusion criteria were age ≥ 18 years, diagnosis of type 1 diabetes for at least one year, stable insulin treatment, defined as no change > 20% of insulin total daily dose in the last three months, use of real-time CGM, and CGM wear time ≥ 70% [18]. Exclusion criteria were the presence of any retinal abnormality related to diabetes, laser photocoagulation, other nondiabetic retinal diseases (including retinal vascular diseases, vitreoretinal diseases, history of central serous retinopathy, or macular dystrophies), ocular media opacity, any previous eye surgical intervention, poor quality of images automatically detected by the instrument including artifact, inaccurate or incorrect segmentation at the level of the SCP and DCP, subject's inability to abstain from blinking or movement during image acquisition, overt diabetic complications (nephropathy, neuropathy, cardiovascular diseases), and other severe comorbidities. A blood sample was taken in all patients with diabetes to measure HbA1c by high-performance-liquid-chromatography (HPLC) aligned with the Diabetes Control and Complications Trial (DCCT) standard. Clinical data were collected from electronic medical records. The dilated slit lamp fundus biomicroscopy was performed after eligible patients gave informed consent to evaluate the exclusion criteria. A control group comparable for sex and age was also enrolled among medical students and medical staff to assess if OCTA variables differed between patients with diabetes and controls without diabetes.

### 2.2. Fundoscopy and OCTA

Each patient underwent dilated slit lamp fundus biomicroscopy before OCTA to evaluate the presence of DR. A skilled operator performed OCTA scans in the morning to avoid possible diurnal variation. OCTA was conducted using XR Avanti AngioVue OCTA (Optovue, Fremont, California, USA). This system uses a split-spectrum amplitude-decorrelation angiography (SSADA) algorithm and operates at 70,000 A-scans per second using a light source of 840 nm. SSADA detects variations in reflected OCT signal amplitudes between two consecutive scans [19, 20]. Decorrelation is a mathematical function that quantifies this variation. SSADA splits the OCT signal into different spectral bands, thus increasing the number of usable image frames, in which each undergoes a decorrelation analysis [19, 20]. Blood flowing through vessels causes a change in reflectance over time and results in localized areas of flow decorrelation between frames. Each scan consisted of 304 × 304 A-scans with two consecutive B-scans at each fixed position. Each scan consisted of one orthogonal horizontal and vertical scan to reduce motion artifacts [19, 20].

We performed OCTA 3 × 3 mm scanning area focused on the fovea centralis. The instrument software automatically segmented OCTA scans into four enface slabs: the SCP, the DCP, the outer retinal plexus, and the choriocapillaris plexus. The software detected the perfused vessel structures within an inner offset at −3 to −15 *μ*m from the inner limiting membrane (ILM) for the evaluation of SCP and within an inner offset at −15 to −70 *μ*m from the ILM for the DCP. The instrument software automatically analyzes SCP and DCP VD in the whole image, foveal, and parafoveal zone. VD was defined as the percentage of blood flow signal within a designated area. Foveal zone VD was defined as the area with a diameter of 1 mm; parafoveal zone VD was defined as the area with a diameter of 3 mm. In this study, we focused on SCP and DCP plexuses and collected for each patient SCP and DCP VD of the whole image, foveal, and parafoveal zone expressed in mm^2^ in the retina plexus. Structural OCT was performed using RTVue OCT (Optovue Inc., Fremont, CA, USA), a high-speed and high-resolution OCT device with a central wavelength of 840 nm and a scan rate of 26,000 A-scans/s and axial resolution of 5 *μ*m. We used the three-dimensional macular scan protocol set to 7 × 7 mm containing 101 horizontal line scans, each consisting of 513 A-scans, to automatically calculate central retinal thickness (CRT) and parafoveal thickness (PFT).

### 2.3. CGM-Derived Glucose Metrics

Glucometric parameters (TIR, TBR, TAR, 24-hour average glucose, CV) of the last 2 weeks were collected through the dedicated software at the time of the OCTA. TIR was defined as the percentage of the time spent in the 70-180 mg/dL glucose range, TBR < 70 mg/dL, and TAR > 180 mg/dL. We also collected the time spent below 54 mg/dL [16].

### 2.4. Statistical Analyses

Normality within glucometric and retinal parameters was visually assessed by means of a quantile-quantile plot. Normally distributed data were described according to mean and SD, while median and quartiles were addressed in not normally distributed data. Absolute and relative frequencies were addressed with categorical variables. Univariate inferential analyses within the two study groups were performed in normal or not normal variables by the *t*-test or the Mann–Whitney *U* test. Proportion tests were issued to assess differences between frequencies. In all instances, a significance level of alpha 0.05 was assumed. Descriptive and univariate inferential analyses have been done using the free software JASP (JASP Team, 2021). Possible relations between glucometric and retinal parameters were explored by means of linear mixed-effects models, as implemented in the "lme4" package [21] of the R [22] software.

## 3. Results

Forty-nine consecutive patients with type 1 diabetes and 34 healthy control subjects participated in the study. Clinical characteristics and glucometric parameters of patients with type 1 diabetes are displayed in Table 1. The sample consisted of young adults with suboptimal glucose control.

Table 2 shows the OCTA measurements in patients with diabetes and control subjects. As shown, SCP and DCP VD of the whole image and parafoveal retina were significantly lower in patients than in controls. The difference persisted after the exclusion of smokers and subjects with hypertension and/or hyperlipidemia. At the same time, no differences were observed for the foveal VD in both plexuses between the two groups.

Linear mixed-effects multiple regression analysis was performed between glucometric and retinal parameters of patients with type 1 diabetes, adjusted for age and disease duration, modeling the clustered measurements of the two eyes within each patient as a nested random effect.

None of the OCTA parameters appeared to be significantly correlated to the glucometric parameters except CV, which was directly correlated with foveal VD in SCP (*t* = 2.126; *p* = 0.04) (Table 3).

We performed the linear mixed-effects multiple regression analysis again after the exclusion of smokers and patients with hypertension or hyperlipidemia. The results are illustrated in Table 4. We found a direct and significant correlation between CV, VD (whole, foveal, and parafoveal) in SCP, and foveal VD in DCP.

## 4. Discussion

In young adults with type 1 diabetes in nonoptimal glucose control and without overt DR, capillary plexus density evaluated by OCTA was significantly lower than in control subjects. The reduction in VD was significant only in the parafoveal zone, while the foveal zone was spared.

Other studies have evaluated changes in retinal VD in patients with diabetes. However, differences exist in the cohorts of examined patients. For instance, Pilotto et al. recruited adolescents with and without DR and found a reduced VD only in patients with clinical signs of DR [17]. In line with Pilotto, Golebiewska et al. found no difference in VD between patients with type 1 diabetes without DR and healthy controls [23]. Notably, also in this study, the participants' mean age was lower than ours. Some methodological differences also emerge by comparing the studies. Indeed, Pilotto et al. considered parafoveal and foveal areas when evaluating VD, and Golebiewska et al. divided the retinal plexus into three layers [17, 23].

Conversely, our results are comparable with the research by Simonett et al. and Sousa et al., who described a significant reduction of the parafoveal VD in the DCP and SCP in young adults with type 1 diabetes [24, 25]. The authors suggested that the highly energy-demanding tissue and the complex vascular architecture make the retina susceptible to hyperglycemia-induced damage.

We did not measure the foveal avascular zone (FAZ), which seems to be enlarged only in patients with DR and longer disease duration [26]. Indeed, Scarinci et al. found that the perimeter and the acircularity index of the FAZ were comparable between patients without DR and controls. The author interestingly commented that the decrease in VD represents a diffuse dropout of parafoveal capillaries rather than an enlargement of the FAZ [27].

According to the study's primary outcome, we found a significant and independent correlation between the extent of the foveal SCP VD and GV expressed as the CV of 24-hour average glucose. The higher the CV, the greater the VD. After the exclusion of smokers and patients with hypertension or hyperlipidemia, we further found a correlation between foveal and parafoveal VD in SCP, foveal VD in DCP, and GV. The observational nature of the study does not allow us to make definitive assumptions about the development of retinal damage. However, we hypothesize that increased VD may precede capillary rarefaction in patients with diabetes, while early rarefaction is mostly correlated to hypertension [28]. The increased VD in diabetes has also been reported by Rosen et al. The authors found that patients without DR had an increased VD compared to control subjects, while patients with nonproliferative and proliferative DR had a reduced VD compared to controls. Accordingly, Rosen et al. defined the increased VD as the “tipping point” of DR [29].

In our study, the mean CV was higher than the target value (40% vs. 36%) in the participants with type 1 diabetes. Since mean TIR was close to 70% and mean TBR was within the recommended threshold of 4%, we hypothesize the high CV to be associated with postprandial hyperglycemia. Indeed, postprandial hyperglycemia is the primary abnormality in case of mild alteration of glucose control, e.g., when the HbA1c is between 7 and 7.5% [30].

Glycemic fluctuations and postprandial hyperglycemic peaks may trigger the increase of local blood flow, at least in the foveal SCP, in analogy to what was reported in other tissues, such as the kidney, muscle, and skin. Hemodynamic changes seem to be mediated by the increased nitric oxide activity and vasodilatory prostaglandins due to local retinal acidosis, which is prominent in the early stages of DR [31–34].

We did not find any other significant correlation between OCTA capillary density and CGM-derived glucose metrics, but conflicting results emerge from the literature about glycemic control and OCTA abnormalities. While poor glycemic control, defined as a high HbA1c value or low TIR, predicts the development of DR [35, 36], evidence concerning glycemic control and retinal vascular density detected by OCTA in subjects with diabetes is still inconclusive. Indeed, some authors found an inverse association between high HbA1c value and capillary plexus, while others did not [10–14]. To our knowledge, the paper by Pilotto et al. is the only one designed to explore the association between the new glycemic indices and OCTA changes in diabetes. Pilotto et al. found a direct relation between TIR and the thickness of the intermediate capillary plexus [17]. However, the authors measured the CGM-derived metrics in a three-month interval, while we used the 2-week one as suggested by the international consensus of experts [16].

## 5. Conclusion

The results of the present study show that, in young adults with type 1 diabetes and without DR, high GV, which can be assessed very accurately by CGM, is associated with increased foveal and parafoveal VD in SCP and with foveal VD in DCP, and that VD in the parafoveal area is reduced in comparison with nondiabetic control subjects. These findings suggest the need for longitudinal studies to clarify the role of these differences in the possible development of DR.

The progressive and indeed rapid refinement of techniques for assessing the retinal vasculature, coupled with the increasingly accurate continuous assessment of blood glucose, give hope that in the near future, we will have reliable markers to follow for the prevention of the development of diabetic retinopathy.

## Figures and Tables

**Table 1:** Clinical characteristics and CGM-derived glucose metrics of the enrolled patients.

Variable	Type 1 diabetes (*N* = 49)
Age, years	27 [25; 36]
Male sex, *N* (%)	23 (47%)
Disease duration, years	13 [9; 40]
HbA1c, %	7.7 ± 1.0
Hypertension, *N* (%)	5 (10)
Hyperlipidemia, *N* (%)	2 (4)
Smoke habit, *N* (%)	2 (4)
TIR, %	61 [21; 74]
TBR, %	2 [6; 33]
TAR, %	37 [26; 77]
Time below 54 mg/dL, %	0 [0; 4]
CV, %	40 ± 30
Average glucose, mg/dL	165 ± 29

HbA1c: glycated hemoglobin; TIR: time in range; TBR: time below range; TAR: time above range. Data are expressed as median (interquartile range), mean ± standard deviation, and prevalence.

**Table 2:** OCTA parameters measured in patients with type 1 diabetes and control subjects.

Parameters	Type 1 diabetes (*N* = 49)	Control subjects (*N* = 34)	*P* value
Eyes (*N*)	98	68	—
SCP VD (mm^2^)			
Whole image	43.8 ± 0.4	47.1 ± 0.7	**<0.0001**
Foveal	18.6 ± 0.8	18.5 ± 1.0	0.46
Parafoveal	46.1 ± 0.5	50.0 ± 0.7	**<0.0001**
DCP VD (mm^2^)			
Whole image	49.2 ± 0.4	51.4 ± 0.7	**0.001**
Foveal	34.7 ± 0.9	36.1 ± 1.1	0.35
Parafoveal	51.1 ± 0.4	53.2 ± 0.7	**0.002**

VD: vessel density; SCP: superficial capillary plexus; DCP: deep capillary plexus. Data are expressed as mean ± standard deviation.

**Table 3:** Correlation between OCTA parameters and CGM-derived glucose metrics in type 1 diabetes.

	OCTA (vascular parameter)	Correlation
TIR (%)	SCP VD Foveal SCP VD Parafoveal SCP VD Whole DCP VD Foveal DCP VD Parafoveal DCP VD	*t* = 0.189; *p* = 0.85 *t* = −0.754, *p* = 0.46 *t* = 0.056, *p* = 0.96 *t* = −0.179, *p* = 0.86 *t* = −0.614, *p* = 0.54 *t* = −0.051, *p* = 0.96

TBR (%)	SCP VD Foveal SCP VD Parafoveal SCP VD Whole DCP VD Foveal DCP VD Parafoveal DCP VD	*t* = 0.48; *p* = 0.63 *t* = 1.46, *p* = 0.15 *t* = −0.128, *p* = 0.90 *t* = −1.165, *p* = 0.25 *t* = −1.667, *p* = 0.10 *t* = 1.148, *p* = 0.26

Average glucose (mg/dL)	SCP VD Foveal SCP VD Parafoveal SCP VD Whole DCP VD Foveal DCP VD Parafoveal DCP VD	*t* = 1.545, *p* = 0.13 *t* = −0.137, *p* = 0.89 *t* = −0.570, *p* = 0.57 *t* = −0.328, *p* = 0.74 *t* = 0.095, *p* = 0.92 *t* = −0.484, *p* = 0.63

CV (%)	SCP VD Foveal SCP VD Parafoveal SCP VD Whole DCP VD Foveal DCP VD Parafoveal DCP VD	*t* = 0.874, *p* = 0.39 *t* = 2.126, *p* = 0.04 *t* = 1.464, *p* = 0.15 *t* = 0.431, *p* = 0.67 *t* = 1.373, *p* = 0.18 *t* = 0.307, *p* = 0.76

VD: vessel density; SCP: superficial capillary plexus; DCP: deep capillary plexus.

**Table 4:** Correlation between OCTA parameters and CGM-derived glucose metrics in type 1 diabetes after exclusion of smokers and patients with hypertension or hyperlipidemia.

	OCTA (vascular parameter)	Correlation
TIR (%)	Whole SCP VD Foveal SCP VD Parafoveal SCP VD Whole DCP VD Foveal DCP VD Parafoveal DCP VD	*t* = 0.746; *p* = 0.46 *t* = −1.158, *p* = 0.25 *t* = 0.195, *p* = 0.85 *t* = −0.187, *p* = 0.85 *t* = −1.513, *p* = 0.14 *t* = 0.007, *p* = 0.99

TBR (%)	Whole SCP VD Foveal SCP VD Parafoveal SCP VD Whole DCP VD Foveal DCP VD Parafoveal DCP VD	*t* = 0.370; *p* = 0.71 *t* = 1.438, *p* = 0.16 *t* = −0.063, *p* = 0.95 *t* = −1.161, *p* = 0.25 *t* = −1.600, *p* = 0.11 *t* = 1.122, *p* = 0.27

Average glucose (mg/dL)	Whole SCP VD Foveal SCP VD Parafoveal SCP VD Whole DCP VD Foveal DCP VD Parafoveal DCP VD	*t* = −0.664, *p* = 0.52 *t* = −0.468, *p* = 0.64 *t* = −0.245, *p* = 0.81 *t* = −0.296, *p* = 0.77 *t* = −0.611, *p* = 0.55 *t* = −0.517, *p* = 0.61

CV (%)	Whole SCP VD Foveal SCP VD Parafoveal SCP VD Whole DCP VD Foveal DCP VD Parafoveal DCP VD	*t* = 2.104, *p* = 0.04 *t* = 3.501, *p* = 0.001 *t* = 2.198, *p* = 0.03 *t* = 1.413, *p* = 0.16 *t* = 2.721, *p* = 0.009 *t* = 1.448, *p* = 0.26

VD: vessel density; SCP: superficial capillary plexus; DCP: deep capillary plexus.

## Data Availability

The data presented in this study are available on request from the corresponding author. Most of the data are sensitive and not publicly available.
